# Carcinoembryonic Antigen in Whole Serum

**DOI:** 10.1038/bjc.1972.46

**Published:** 1972-10

**Authors:** J. M. MacSween, N. L. Warner, A. D. Bankhurst, I. R. Mackay

## Abstract

A microradioimmunoassay technique is described for detecting carcinoembryonic antigen (CEA) in whole serum. It differs from previous methods in being performed on 0·025 ml of whole serum instead of 5 ml of serum extracted with perchloric acid. The present assay was sufficiently sensitive to detect 85% of carcinomata, localized to the colon, but positive results occurred also with certain non-gastrointestinal cancers, chiefly lung and breast, and certain non-malignant diseases. Many of the latter sera, with the general exception of alcoholic cirrhosis and pancreatitis, gave negative results after extraction with perchloric acid. It is suggested that a direct assay for CEA in whole serum may permit testing of large numbers of sera by laboratories with facilities for radioimmunoassays.


*Br. J. Cancer* (1972) **26**, 356

### CARCINOEMBRYONIC ANTIGEN IN WHOLE SERUM

J. M. MacSWEEN*, N. L. WARNER, A. D. BANKHURST† and I. R. MACKAY

*From the Clinical Research Unit and the Laboratory of Immunogenetics of the Walter and Eliza Hall Institute of Medical Research, and the Royal Melbourne Hospital, Victoria* 3050, *Australia*

Received 9 May 1972. Accepted 12 June 1972

**Summary.—A microradioimmunoassay technique is described for detecting carcinoembryonic antigen (CEA) in whole serum. It differs from previous methods in being performed on 0·025 ml of whole serum instead of 5 ml of serum extracted with perchloric acid. The present assay was sufficiently sensitive to detect 85% of carcinomata, localized to the colon, but positive results occurred also with certain non-gastrointestinal cancers, chiefly lung and breast, and certain non-malignant diseases. Many of the latter sera, with the general exception of alcoholic cirrhosis and pancreatitis, gave negative results after extraction with perchloric acid. It is suggested that a direct assay for CEA in whole serum may permit testing of large numbers of sera by laboratories with facilities for radioimmunoassays.**

Carcinoembryonic antigen (CEA) is an antigen present in human foetal digestive tissues and in cancer of these tissues in adult life (Gold and Freedman, 1965*a*, 1965*b*; Krupey, Gold and Freedman, 1968). It may also be found in the serum of patients with gastrointestinal cancer. Detection of CEA in serum therefore promises to be of aid in the diagnosis of these cancers. A radioimmunoassay technique for CEA has been developed (Thomson *et al.*, 1969; Moore *et al.*, 1971; LoGerfo, Krupey and Hansen, 1971) which required as an initial step extraction of the antigen from serum with perchloric acid. In this paper we describe a radioimmunoassay for detection of CEA in small samples of whole serum.

### MATERIALS AND METHODS

Sera were obtained from 51 blood donors and from 326 patients selected to include those with various neoplastic and non-neoplastic diseases.

Purified CEA and a goat antiserum to CEA were generously provided by Dr P. Gold, Montreal. Samples (20 μg) of CEA were labelled with $^{125}I$ using chloramine T as oxidant (Hunter and Greenwood, 1962); the specific activity of various preparations was 22–50 μCi/μg. The labelled antigen was diluted in 0·01 mol/l tris buffer, pH 7·5, containing 3% bovine serum albumin (BSA), to a concentration of approximately 1 ng/ml. Antiserum to CEA was diluted in BSA-tris buffer; 25 μl of a $1 \times 10^{-5}$ dilution was used throughout, this giving approximately 30% precipitation of the amount of $^{125}I$ CEA used. Each serum assay was performed in duplicate in 6 × 50 mm tubes by mixing 50 μl of $^{125}I$ CEA with 25 μl of the test human serum and 25 μl of the diluted goat antiserum to CEA; the tubes were held for 2 hours at 37°C and then overnight at 4°C. We assessed 2 alternatives for precipitating complexes of CEA with antiserum to CEA, *i.e.* ammonium sulphate as used in the conventional procedure on extracted serum (Thomson *et al.*, 1969), and antiserum to goat gamma globulin.

Saturated ammonium sulphate (100 μl) was added to each tube, immediately mixed, held for one hour at 4°C, centrifuged at 16,000 *g* for 10 minutes, after which 100 μl samples of the supernate from each tube were removed for counting of radioactivity. Standard tubes included (a) $^{125}I$ CEA with

* Fellow, Medical Research Council of Canada.
† Cleveland Fellow of The Royal Melbourne Hospital.

pooled normal human serum, and (b) $^{125}$I CEA antiserum to CEA and pooled normal human serum. A standard curve was developed by mixing $^{125}$I CEA with antiserum to CEA, normal human serum and doubling dilutions of purified unlabelled CEA at concentrations of 3–100 ng per ml. Inhibition by test serum of precipitation of $^{125}$I CEA with antiserum to CEA was compared with the degree of inhibition given by unlabelled purified CEA, and the concentration of CEA in the test serum accordingly determined; levels of 10 ng/ml were detectable.

In the alternative procedure for precipitation, using rabbit antiserum to goat gamma globulin (Hoechst Australia, Limited), test human serum was held with antiserum to CEA at 37°C for 2 hours and overnight at 4°C before the addition of $^{125}$I CEA. After a further period of 2 hours at 37°C and overnight at 4°C, 100 $\mu$l of a 1 : 20 dilution of rabbit antiserum to goat gamma globulin was added to each tube; tubes were held at 37°C for 2 hours and overnight at 4°C and centrifuged, and supernates were counted as described above, using the same sets of controls. Aspects relating to conditions for antigen–antibody interaction and precipitation of immune complexes will be reported in detail elsewhere. Levels of CEA in serum of 5 ng/ml were detectable.

A third series of assays was carried out to compare results on whole serum with those obtained on sera extracted with perchloric acid. 0·1 ml of 4 mol/l perchloric acid was added to 0·4 ml of serum whilst the serum was agitated on a vortex mixer; the tubes were centrifuged at 4000 *g* for 10 minutes and the supernate dialysed against multiple changes of 0·01 mol/l phosphate buffered saline, pH 7·3, for 2 days; 25 $\mu$l samples of the extract were assayed for CEA, as described, with precipitation of immune complexes by ammonium sulphate. Precipitation was facilitated by adding a $5 \times 10^{-1}$ dilution of normal goat serum to the antiserum to CEA. Control tubes contained perchloric acid extracts of pooled normal human serum. A standard inhibition curve was obtained by including with each assay perchloric acid extracts of pooled normal human serum to which had been added, before extraction, doubling dilutions of unlabelled CEA. When levels of CEA were low, tests were occasionally repeated on supernates concentrated by lyophilization. Levels of CEA in serum of 3 ng per ml were detectable.

### RESULTS

The results of assays for CEA are shown in Tables I and II. The inhibition of the reaction between $^{125}$I CEA–anti CEA produced by the test sera are expressed as ng of CEA per ml of serum relative to the purified CEA standard. The results of assays for CEA in whole serum using precipitation by rabbit anti-goat serum (Table II) were generally similar to those using ammonium sulphate precipitation (Table I). However, more sera gave detectable levels of CEA, particularly sera from patients with

TABLE I.—*Detection of CEA in Whole Serum and in Extracted Serum using Ammonium Sulphate for Precipitation of Immune Complexes*

| | Whole serum | | | | Extracted serum* | | | |
|---|---|---|---|---|---|---|---|---|
| | Number | CEA ng/ml | | | Number | CEA ng/ml | | |
| Diagnosis | tested | < 10 | 10–20 | > 20 | tested | < 3 | 3–15 | > 15 |
| Blood donors | 17 | 16 | 1 | — | 21 | 20 | 1 | — |
| Miscellaneous diseases† | 35 | 31 | 1 | 3 | 43 | 39 | 4 | — |
| Cancer of colon, local | 12 | 9 | 2 | 1 | 17 | 6 | 9 | 2 |
| Cancer of colon, disseminated | 9 | 1 | 1 | 7 | 10 | 2 | 4 | 4 |
| Other gastrointestinal cancer | 28 | 11 | 7 | 10 | 14 | 3 | 7 | 4 |
| Cancer of lung and breast‡ | 31 | 14 | 5 | 12 | 25 | 7 | 11 | 7 |
| Other cancer | 23 | 21 | — | 2 | 9 | 5 | 4 | — |
| Pancreatitis and alcoholic cirrhosis‡ | 12 | 7 | 1 | 4 | 16 | 4 | 11 | 1 |

* Serum extracted with perchloric acid.
† Excluding cancer, pancreatitis and liver disease.
‡ Pooled data—approximately equal proportions for both diseases.

TABLE II.—*Detection of CEA in Whole Serum using Rabbit Antiserum to Goat Gamma Globulin for Precipitation of Immune Complexes*

| Diagnosis | Number tested | CEA ng/ml < 5 | 5–14 | 15–35 | > 35 |
|---|---|---|---|---|---|
| Blood donors | 51 | 46 | 5 | — | — |
| Miscellaneous diseases* | 158 | 102 | 43 | 13 | — |
| Cancer of colon { local | 23 | 3 | 9 | 9 | 2 |
| Cancer of colon { disseminated | 21 | — | 1 | 10 | 10 |
| Other gastrointestinal cancer | 34 | 4 | 10 | 8 | 12 |
| Cancer of lung | 22 | 2 | 10 | 3 | 7 |
| Cancer of breast | 14 | 3 | 3 | 4 | 4 |
| Other cancer | 31 | 26 | 3 | 2 | — |
| Pancreatitis | 13 | 4 | 2 | 6 | 1 |
| Alcoholic cirrhosis | 11 | 1 | 4 | 6 | — |

* Excluding cancer, pancreatitis and alcoholic cirrhosis of the liver.

localized carcinoma of the colon. On the other hand, more sera from patients with diseases other than cancer gave detectable levels of CEA. The results of assays for CEA after extraction of sera with perchloric acid (Table I) show concentrations of CEA lower than those obtained with whole serum; in particular, extracted sera from 6 of 17 patients with localized colonic cancer had a concentration of CEA less than 3 ng/ml. We draw attention to the finding, in all procedures, of CEA in sera of patients with non-intestinal cancers, particularly of the lung and breast, as well as in sera of some patients with diseases other than cancer, particularly cirrhosis of the liver and pancreatitis.

### DISCUSSION

Early studies suggested that CEA was specifically elevated in the sera of patients with gastrointestinal cancer (Thomson *et al.*, 1969). More recent studies, however, have shown that CEA can also be detected in the sera of patients with non-intestinal cancer, particularly of lung and breast, in certain non-neoplastic diseases, particularly of gastro-intestinal or pulmonary origin, and in uraemia (Moore *et al.*, 1971; LoGerfo *et al.*, 1971). Our present results are in accord with these observations, in showing that CEA was detected in association with cancer of the colon, stomach, pancreas, breast and lung, and in lower concentration in some non-malignant diseases. The cases of cancer of the colon were " staged " only to the degree of differentiating " local " and " disseminated " cancer; " local " refers to cancers restricted to the wall of the bowel (Duke's Class 1) and " disseminated " to cancers extending beyond the wall of the bowel (Duke's Class 2 and 3, and distant metastases). Other cancers were not " staged ". We note that sera from 20 of 23 " local " colon cancers were positive, as were all of 21 " disseminated " cancers, using antiglobulin precipitation of complexes (Table II).

In previous studies (Thomson *et al.*, 1969; Moore *et al.*, 1971; LoGerfo *et al.*, 1971), CEA was detected in serum only after extraction with perchloric acid, dialysis and usually lyophilization, using approximately 5 ml of patient serum as opposed to 0·025 ml of whole serum in our modified microassay. However, it is first relevant to compare our results on sera extracted with perchloric acid with those cited in previous reports. The present microassay, using 25 $\mu$l of extracted serum, was sufficiently sensitive that 3 ng/ml of CEA could be detected. With extracted sera, we found that most cases of disseminated carcinoma of the colon, other intestinal cancers and cancer of the lung and breast gave values over 3 ng/ml, although sera from 6 of 17 patients with cancers known to be localized to the colon had no detectable levels.

The present studies were designed to develop a microassay technique which would detect CEA in small volumes of whole serum. This was achieved in assays in which the complexes were precipitated by rabbit antiserum to goat gamma globulin. This proved better than precipitation of complexes by ammonium sulphate; however, we note that the lowest detectable level of CEA was 10 ng/ml using ammonium sulphate. Taking each of our methods into consideration, the highest incidence of positive assays for CEA with normal human sera was 10% (5/51), with levels between 3–10 ng/ml, all other sera having values less than 3 ng/ml. It may be that all human sera contain some CEA, and considerably more work will be required in order to derive a " normal range ".

Most of the sera from patients without cancer which gave levels of CEA over 5 ng/ml in direct tests on whole serum were re-assayed after extraction with perchloric acid in order to reassess these apparently " false positive " results. The assay remained positive with many sera of patients with alcoholic cirrhosis of the liver and pancreatitis; it is assumed that such sera do in fact contain CEA. Of the 4 positive sera from patients with miscellaneous diseases, extraction resulted in a reduction in the level of CEA, from greater than 20 ng/ml to less than 15 ng/ml. Negative results in assays for CEA using whole serum from patients with known cancer of the colon remained negative after extraction. Hence, we conclude that although a satisfactorily sensitive microimmunoassay technique for detecting CEA in whole serum was attained, " false positive " results occur in some conditions other than cancer, so that positive sera should be re-assayed after extraction with perchloric acid; conversely, negative results may occur in small cancers localized in the colon. To validate further this conclusion, a larger series of sera from patients with cancers and other diseases is being processed by all 3 assay methods.

The assays on whole serum reported here were carried out by a micro-method requiring only 25 $\mu l$ of serum. Such assays can therefore be performed on fresh or stored sera drawn for other purposes, and many determinations can be made on the one sample. Since this assay merely requires the addition of reagents, centrifugation and sampling of supernates, it should be readily adaptable to automated techniques. Although further studies on specificity of the assay are required and additional modifications may be needed to reduce the frequency of " false positive " results in relation to cancer, the assay for CEA in whole serum appears applicable to the testing of large numbers of sera by routine laboratories with facilities for radioimmunoassays.

We are indebted to Dr Phil Gold for providing CEA and antiserum to CEA, to Mrs Dorothy Goriup for excellent technical assistance and to Dr Rob Burton and Sister I. Langford for assistance in obtaining serum. This work was supported by research grants from the Bushell Trust and the National Health and Medical Research Council of Australia. This is publication No. 1716 from the Walter and Eliza Hall Institute of Medical Research.

### REFERENCES

GOLD, P. & FREEDMAN, S. O. (1965*a*) Demonstration of Tumour-specific Antigens in Human Colonic Carcinomata by Immunological Tolerance and Absorption Techniques. *J. exp. Med.*, **121**, 439.

GOLD, P. & FREEDMAN, S. O. (1965*b*) Specific Carcinoembryonic Antigens of the Human Digestive System. *J. exp. Med.*, **122**, 467.

HUNTER, W. M. & GREENWOOD, F. C. (1962) Preparation of Iodine-131 Labelled Human Growth Hormone of High Specific Activity. *Nature, Lond.*, **194**, 495.

KRUPEY, J., GOLD, P. & FREEDMAN, S. O. (1968) Physicochemical Studies of the Carcinoembryonic Antigens of the Human Digestive System. *J. exp. Med.*, **128**, 387.

LOGERFO, P., KRUPEY, J. & HANSEN, H. (1971) Demonstration of an Antigen Common to Several Varieties of Neoplasia. *New Engl. J. Med.*, **285**, 138.

MOORE, T. L., KUPCHIK, H. Z., MARCON, N. & ZAMCHECK, N. (1971) Carcinoembryonic Assay in Cancer of the Colon and Pancreas and Other Digestive Tract Disorders. *Am. J. dig. Dis.*, **16**, 1.

THOMSON, D., KRUPEY, J., FREEDMAN, S. O. & GOLD, P. (1969) The Radioimmunoassay of Circulating Carcinoembryonic Antigen of the Human Digestive System. *Proc. natn. Acad. Sci. U.S.A.*, **64**, 161.


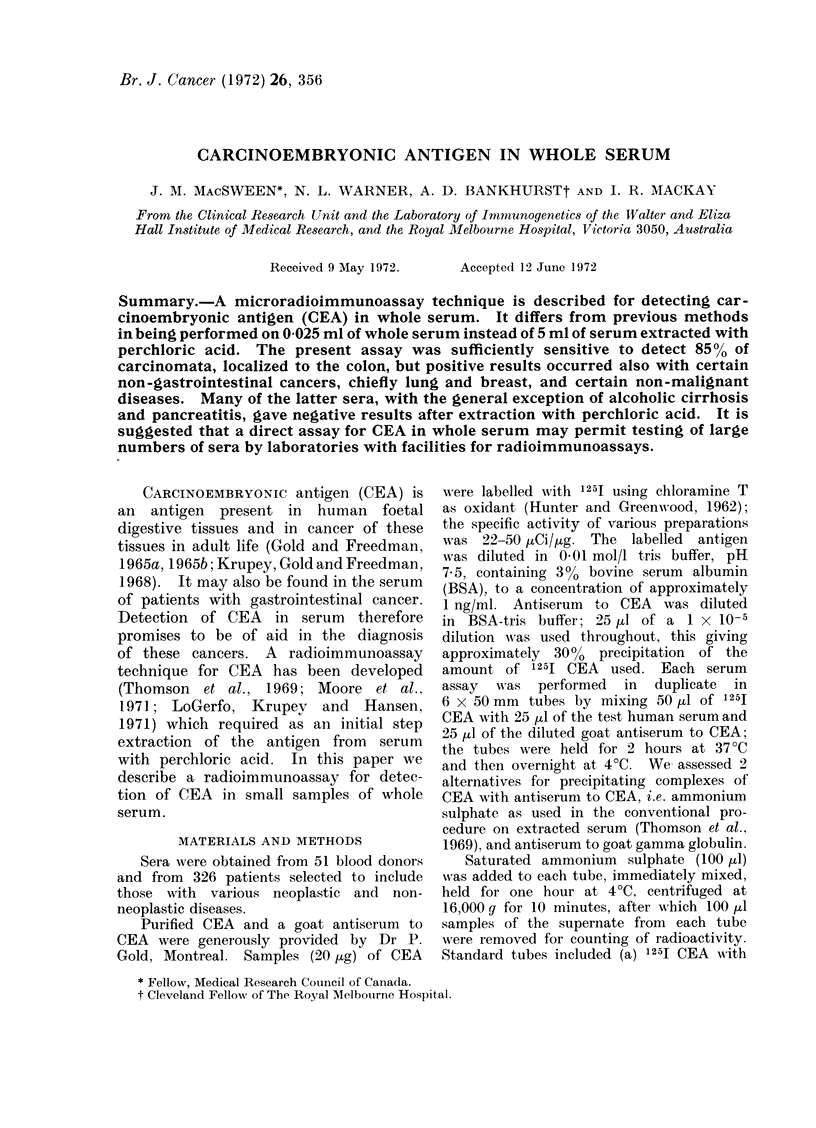

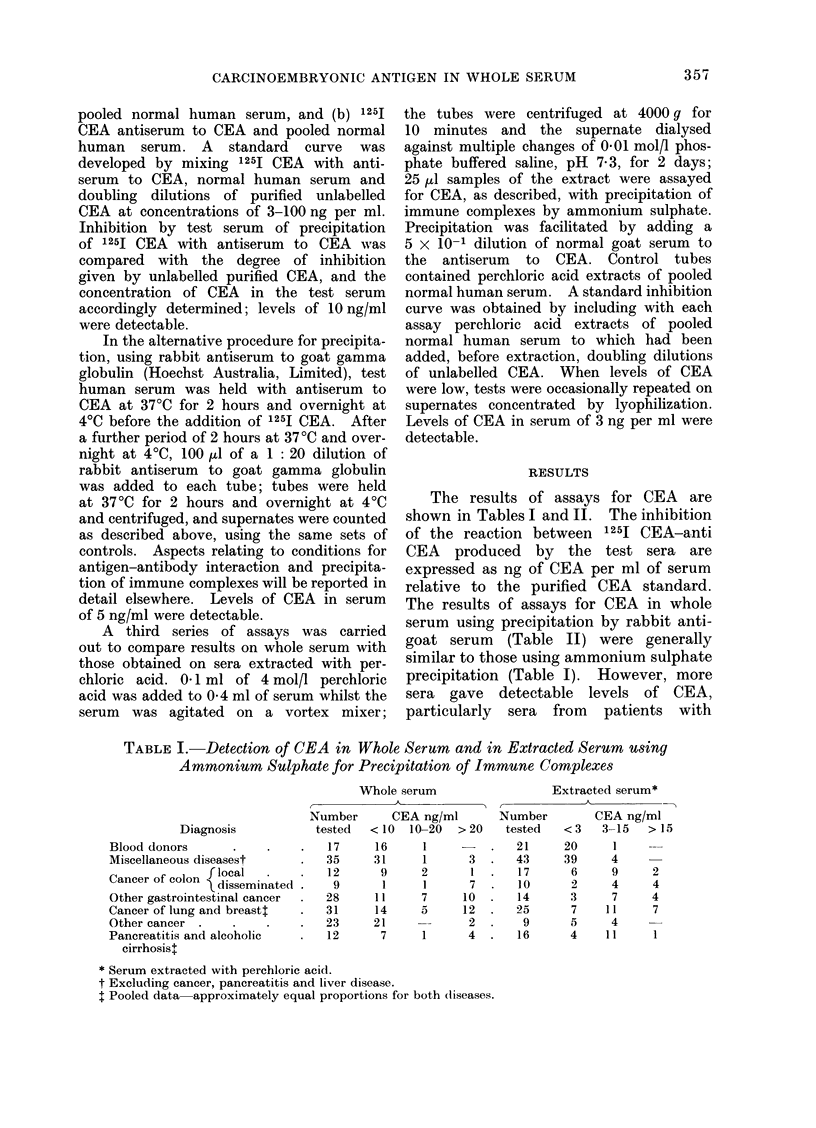

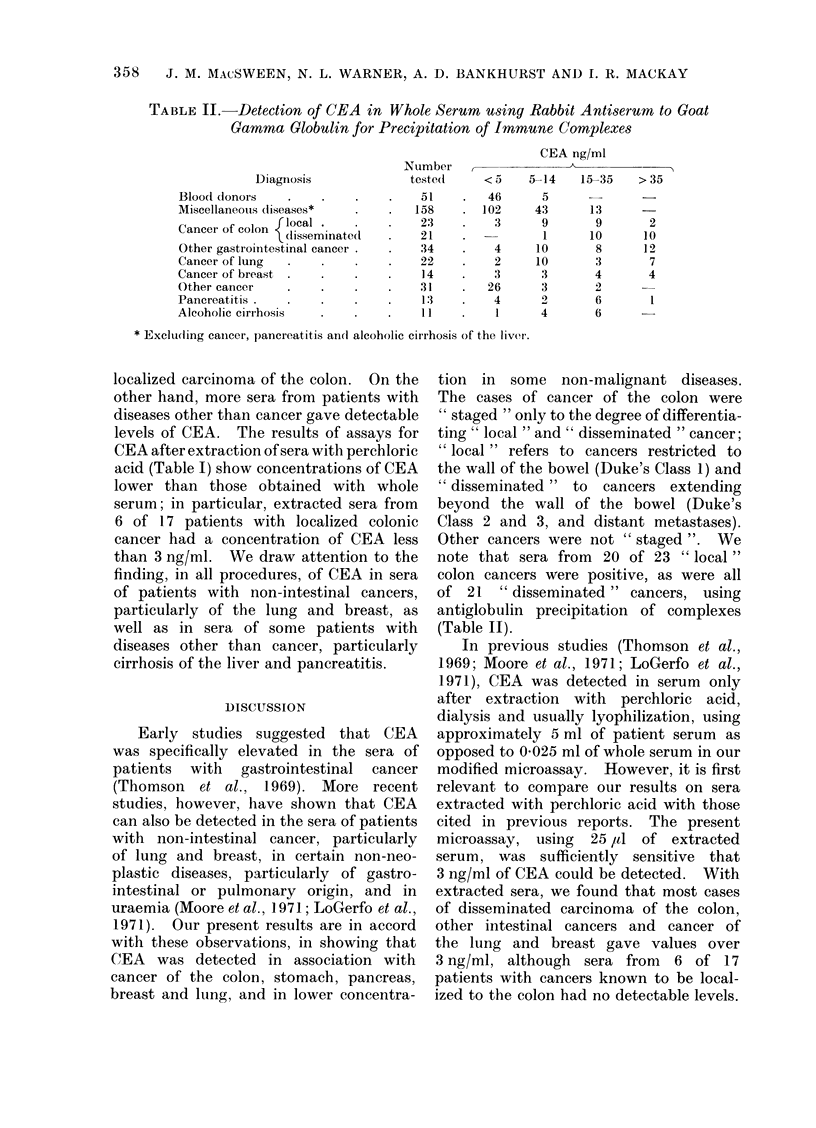

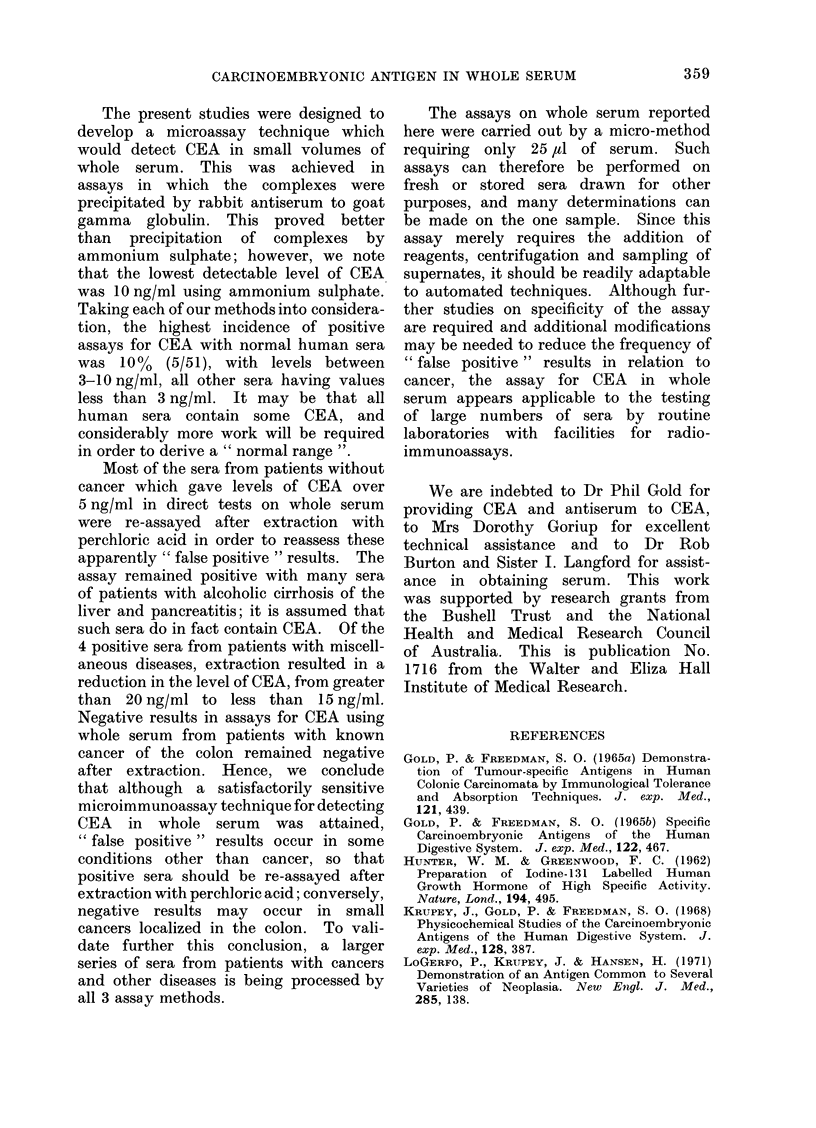

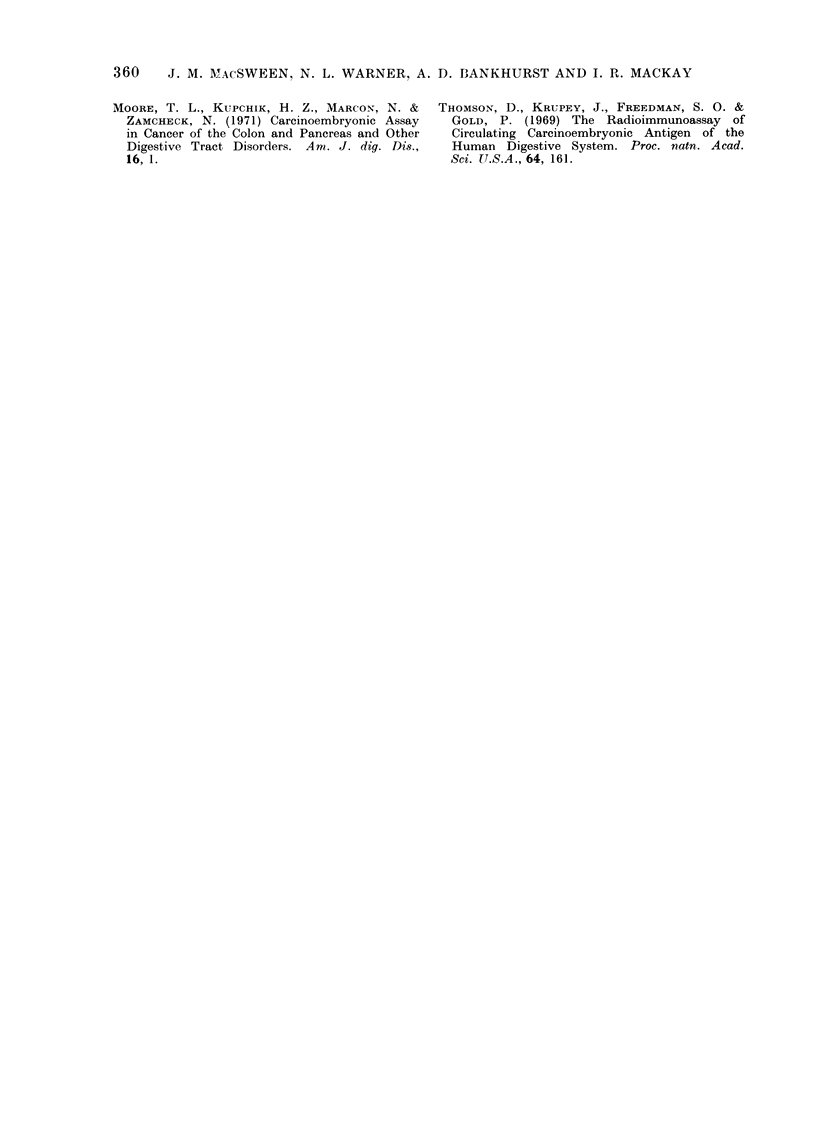


## References

[OCR_00478] GOLD P., FREEDMAN S. O. (1965). DEMONSTRATION OF TUMOR-SPECIFIC ANTIGENS IN HUMAN COLONIC CARCINOMATA BY IMMUNOLOGICAL TOLERANCE AND ABSORPTION TECHNIQUES.. J Exp Med.

[OCR_00485] Gold P., Freedman S. O. (1965). Specific carcinoembryonic antigens of the human digestive system.. J Exp Med.

[OCR_00490] HUNTER W. M., GREENWOOD F. C. (1962). Preparation of iodine-131 labelled human growth hormone of high specific activity.. Nature.

[OCR_00496] Krupey J., Gold P., Freedman S. O. (1968). Physicochemical studies of the carcinoembryonic antigens of the human digestive system.. J Exp Med.

[OCR_00502] Lo Gerfo P., Krupey J., Hansen H. J. (1971). Demonstration of an antigen common to several varieties of neoplasia.. N Engl J Med.

[OCR_00512] Moore T. L., Kupchik H. Z., Marcon N., Zamcheck N. (1971). Carcinoembryonic antigen assay in cancer of the colon and pancreas and other digestive tract disorders.. Am J Dig Dis.

[OCR_00517] Thomson D. M., Krupey J., Freedman S. O., Gold P. (1969). The radioimmunoassay of circulating carcinoembryonic antigen of the human digestive system.. Proc Natl Acad Sci U S A.

